# Can telemedicine reach rural, older veterans on the edge of or caught in the digital divide? – Unique considerations for two distinct populations

**DOI:** 10.1080/28324897.2024.2336899

**Published:** 2024-04-01

**Authors:** Kathryn A. Nearing, Eileen M. Dryden, Camilla B Pimentel, Laura M. Kernan, Stephanie Hartz, Lynette Kelley, Hillary D. Lum, William W. Hung, Meaghan A. Kennedy, Lauren R. Moo

**Affiliations:** a Eastern Colorado Geriatric Research Education and Clinical Center, VA Eastern Colorado Healthcare System, Aurora, CO, USA; b Division of Geriatric Medicine, University of Colorado, Aurora, CO, USA; c Center for Healthcare Organization and Implementation Research, Department of Veterans Affairs (VA) Bedford Healthcare System, Bedford, MA, USA; d New England Geriatric Research, Education, and Clinical Center, VA Bedford Healthcare System, Bedford, Massachusetts, USA; e Department of Orthopaedics, Dartmouth-Hitchcock Medical Center, New Hampshire, Lebanon; f Denver-Seattle Center of Innovation for Veteran-Centered and Value-Driven Care (COIN), Rocky Mountain Regional VA Medical Center, Aurora, CO, USA; g Bronx Geriatric Research Education and Clinical Center, James J. Peters VA Medical Center, Bronx, NY, USA; h Department of Geriatrics and Palliative Medicine, Icahn School of Medicine, New York City, New York, USA; i Department of Family Medicine, Boston University School of Medicine, Boston, Massachusetts, USA; j Department of Neurology, Harvard Medical School, Boston, MA, USA

**Keywords:** Telemedicine, rural, Veterans, digital divide, social determinants of health, geriatrics

## Abstract

GRECC Connect, a national program with interprofessional teams at urban-based VA medical facilities, partner with VA community-based outpatient clinics (CBOCs) to provide geriatric specialty care via telemedicine to rural, older Veterans. Our QI project explored factors affecting program uptake. February-May 2020 we conducted 50 interviews with CBOC staff across the US; 60–80% of patients were rural/highly rural older Veterans. CBOC staff described social determinants of health negatively impacting telemedicine access. Patients on the edge of the digital divide were at risk of diminished access due to changes in physical, cognitive or emotional health and/or socio-economic status. CBOC staff also described highly rural Veterans caught in the digital divide, without access to reliable internet, devices or computer knowledge/skills; included in this subgroup were Veterans staff described as ‘off the grid’ due to histories of trauma resulting in mental/physical health challenges, distrust of institutions and technology, and desire for geographic/social isolation. This work differentiated rural, older Veterans GRECC Connect served through telemedicine, from those CBOC partners struggled to reach, even by phone. This digital divide may grow given the aging population. Unique contextual factors influencing telemedicine use among older adult populations are important to elucidate to inform structural supports for enhanced access.

## Introduction

The US Department of Veterans Affairs (VA) is the largest healthcare system in the United States and arguably the healthcare system with the largest mandate. This excerpt, which frames the VA 2018–2024 strategic plan, states:

The … VA workforce [is] dedicated to treating those Americans who so willingly volunteered their lives in defense of this great Nation. **VA pledges to** advocate and **provide care for all eligible Veterans** who come to us, **with emphasis on those who will need us the most but have the least ability to reach out to us for help. VA will ensure that our most vulnerable Veterans are cared for**. (US Department of Veterans Affairs). [page 7; emphasis added]

This excerpt not only highlights the VA’s commitment to serving all Veterans but also the dynamic interplay between access to care and care needs, with those who have the most complex needs often having the least access to care.

With the VA’s mission as a backdrop, this paper presents themes from 50 key informant interviews with staff from multiple professions who serve rural and highly rural, medically complex, older Veterans; these staff utilize telemedicine as one modality for extending access to geriatric specialty care. Findings surfaced two key tensions:

1. The tension between the mission to serve all Veterans and the difficulty of reaching highly rural, older Veterans who are socially, economically and technologically isolated, often as a direct or indirect result of their service; and,
2. The tension between promoting telemedicine to expand access to care and the difficulties of rural, older Veterans accessing telemedicine due to the intersectionality of geography, age, and social risk factors.

We argue that the dynamic interplay of these conditions gives rise to the digital divide as yet another salient social determinant of health for rural, older Veterans (Clare, 2021; Sieck et al., 2021). In this paper, we use the term digital divide to refer to disparities in access to adequate broadband infrastructure, reliable internet connectivity, up-to-date technology equipment, and digital or computer literacy, with disparities impacted by health conditions (mental health, physical and cognitive functioning), age, geography, and social risk factors such as education and job opportunities. (This and other key terms used in this paper are defined in Appendix A: Glossary of Terms.) Our findings document rural telemedicine practitioner experiences trying to reach rural, older Veterans on the edge of, or caught in, the digital divide. Resulting insights add more credence to the emerging conceptualization of digital access as a super determinant of health—one that underlies and/or exacerbates other social, economic and environmental conditions affecting health (County Health Rankings and Roadmaps, 2021; Federal Communications Commission [FCC], 2022; Turcios, 2023).

### Rural US veterans

About one in four, or 4.4 M, US Veterans reside in a rural or highly rural area, as determined by Rural Urban Commuting Area (RUCA) codes (US Department of Agriculture Economic Research Service, 2023; US Department of Veterans Affairs Office of Rural Health, 2023). (The VA classifies by census tracts. Census tracts with RUCA scores of 1.0 or 1.1 are designated Urban (U). Census tracts with RUCA scores of 10.0 are designated Highly Rural (H or HR). All other census tract RUCA scores are designated Rural (R).) The VA Office of Rural Health reports that, ‘61 percent of rural Veterans are enrolled in the VA healthcare system—significantly higher than the 41 percent enrollment rate of urban Veterans’ (US Department of Veterans Affairs Office of Rural Health, 2023). The VA dedicates close to a third of its annual budget to caring for rural Veterans (US Department of Veterans Affairs Office of Rural Health, 2023), with $39 M invested in internet connectivity and equipment to expand access to telemedicine services in 2020 in response to the COVID-19 pandemic (Landi, 2020). According to the Office of Rural Health 2020 Annual Report, ‘Nearly half of VA telemedicine patients connect from rural areas’ (page 7) (U.S. Department of Veterans Affairs Veterans Health Administration Office of Rural Health).

Despite the VA’s investment in digital inclusion and the uptake of telemedicine among some rural Veterans, the demographics of rural, older Veteran populations are highly correlated with digital disparities. The Digital Divide Index (DDI) quantifies digital disparities among populations. This composite measure combines an infrastructure/adoption score comprised of 5 variables (e.g. percent of homes with internet access and computers/digital equipment) with a socio-economic score, also comprised of five variables (e,g., percentage of the population ages 65 and over, percentage of the population 25 and over with less than high school education, and percentage of noninstitutionalized civilian population with a disability) (Gallardo, 2023). The Purdue University Center for Regional Development applied the DDI to identify US counties with a high, moderate or low DDI. Their findings highlight variability by geography and note that, ‘a higher share of the population in counties with a high digital divide are rural, veterans, living in poverty, and disabled’ (page 3) (Gallardo, 2022).

The work of Darrat and colleagues (Darrat et al., 2021) from March through May 2020 – a period that corresponds with the time during which we conducted our key informant interviews—also highlights demographic characteristics that correlate with the digital divide. Specifically, Daratt et al. conducted a cohort study of 1,162 individuals during the initial COVID-19 surge and reported disparities in access to telemedicine among men, those of lower socio-economic status, the un- or underinsured, older adults, and individuals who were not married.

Table 1 shows that, compared to urban Veterans, rural Veterans are more likely to have demographic characteristics highly correlated with disparities in digital access identified in this previous work (i.e. research applying the DDI and Darrat et al.). To understand the magnitude of the corresponding disparities, the Purdue University report, ‘The State of the Digital Divide in the United States’, is instructive. Using data from the US Census Bureau 5-year American Community Survey, the author reported that households with an annual income of $35,000 or less were nearly 20 percent more likely to be in a high digital divide county (page 5) (Gallardo, 2022). The VA Office of Rural Health reports that 44 percent of rural Veterans earn less than $35,000 annually (US Department of Veterans Affairs Office of Rural Health, 2023).

These data highlight the potential scope and magnitude of the digital divide as a social determinant of health among rural, older Veterans. The extensive literature on the social determinants of health substantiates that structural conditions systematically disadvantage some populations, such as racial/ethnic minoritized populations, rural communities and older adults, impacting poorer health both directly and indirectly, through lack of access to quality healthcare services. Studies examining the rapid transition to telemedicine during the COVID-19 pandemic suggest this shift may have widened access to care gaps for rural, older and socioeconomically disadvantaged Veterans with significant health challenges (Dhanani et al., 2023; Leung et al., 2023; Tisdale et al., 2024). Our key informant interviews with community-based outpatient clinic (CBOC) staff allowed us to explore their experiences in trying to address the VA mandate to serve all who served within this digital divide context.

## Materials and methods

### GRECC connect national initiative to expand access to geriatric specialty care

GRECC Connect is a national initiative funded by the VA Office of Rural Health, the goal of which is to increase access to geriatric specialty care for rural older Veterans with multiple chronic conditions and complex care needs (Pimentel et al., 2019). GRECC Connect interprofessional teams most often include geriatricians, nursing professionals, social workers, pharmacists, psychologists, physical therapists and occupational therapists who constitute a GRECC Connect ‘hub’ working from an urban-based VA medical center. GRECC Connect has expanded the number of hub sites from 4 initial hubs in 2014 to 18 hubs currently (Geriatric Scholars Virtual Learning Community, 2023; Hung et al., 2014). The most common GRECC Connect services include geriatric assessment, dementia diagnosis and management, medication review and reconciliation, fall risk assessment/rehabilitation, mental health care, goals of care/Advance Care Planning, and caregiver support.

VA community-based outpatient clinics are VA satellite clinics that provide the most common outpatient healthcare services closer to where rural Veterans live. CBOCs are typically staffed by a primary care clinician, pharmacist, psychologist, social worker, nurse professional, telehealth technician and clinic manager. Services vary by CBOC but typically include primary care, mental health care, and management of chronic healthcare conditions. Telemedicine is used to extend access to specialty care not available onsite. GRECC Connect interprofessional teams support CBOC staff in caring for older patients using three strategies: 1) education and e-consultations with CBOC staff, 2) telemedicine appointments with Veterans at CBOC locations, and 3) telemedicine appointments to Veteran’s homes (Hung et al., 2014; H. D. Lum et al., 2020; Pimentel et al., 2019).

### Quality improvement project to identify and address barriers to reaching rural and highly rural older veterans with complex care needs

**Figure 1.**
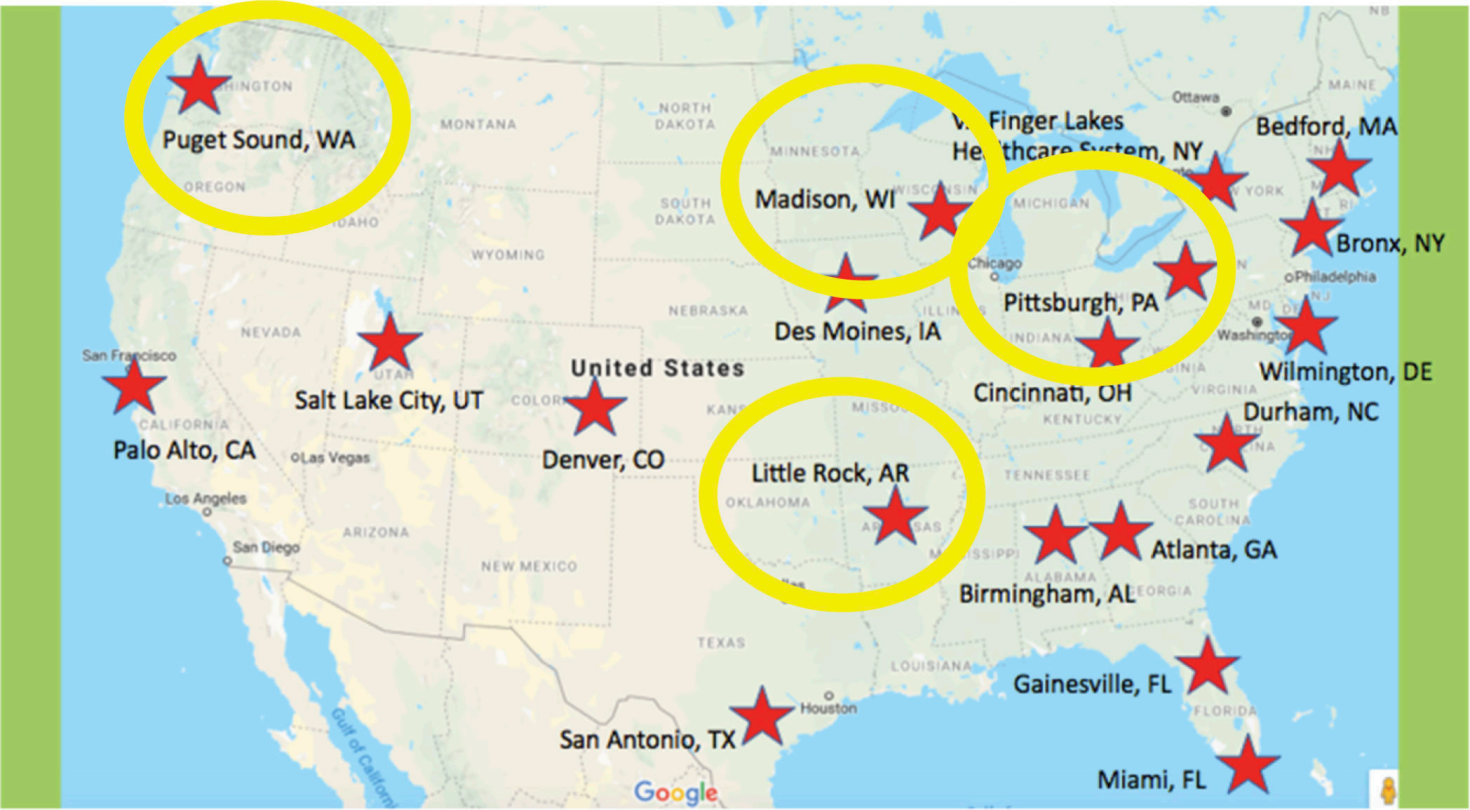
National sample of community-based outpatient clinic staff (*n* = 50) affiliated with GRECC connect hub sites on east and west coast, as well as the south and mid-west regions of US.

February through May 2020, the national GRECC Connect evaluation core conducted 50 key informant interviews with staff who worked in VA-affiliated CBOCs in the regions highlighted in Figure 1. The methodology for selecting CBOCs involved a multi-step process and is described in detail in the original publication (Pimentel et al., 2023). Briefly, we identified GRECC Connect hubs with the largest rural catchment areas. Recognizing that social risk factors vary by location, we purposively selected four GRECC Connect hubs to maximize the geographic variation in our sample (Northeast, Southeast, Midwest and Western US). Once the GRECC hubs were identified, we worked with hub site leads to select four rural CBOCs per hub that varied in their utilization of GRECC Connect services (16 CBOCs, total). We initiated recruitment of associated CBOC staff for interviews through clinical managers who provided CBOC staff contact information. In recruiting staff to interview, we prioritized those most likely to refer patients to GRECC Connect and/or coordinate telemedicine visits for qualifying older Veterans. Four evaluators (KAN, ED, CP, LK) created the semi-structured key informant interview guide (Appendix B) and conducted the 1-hour phone interviews. The purpose of these key informant interviews was to explore contextual factors, as well as factors at the clinic and patient levels, that contributed to or impeded uptake of GRECC Connect services. This work was reviewed by an institutional review board and determined to be quality improvement (not human subjects research). Interviews were recorded with permission, professionally transcribed, checked for accuracy, and coded using a framework analysis approach (Gale et al., 2013; Ritchie & Spencer, 2022). We used questions from the key informant interview guide as the structure for organizing data extracted from transcripts. We then examined patterns (themes) across key informant responses in relation to each question. More details regarding the methodology and population, as well as general themes from the interviews, have been detailed previously (Pimentel et al., 2023).

## Results

### Rurality of participating CBOCs

Staff from 13 CBOCs, affiliated with four hub sites at urban-based medical centers, were interviewed. Seven of the 13 participating CBOCs were located in rural areas (RUCA = >1.1), and one was located in a highly rural area (RUCA = 10) as defined by the RUCA tool for rurality developed by the Department of Agriculture (US Department of Agriculture Economic Research Service, 2023; Washington State Department of Health, 2016). All served significant percentages of rural Veterans (average 72%; sd 26) (US Department of Veterans Affairs).

### Findings: Rural clinic staff describe key characteristics of the rural, older veterans they try to reach and serve

In line with objective data available for participating CBOCs, staff we interviewed consistently reported that between 60–80% of their patient panels could be characterized as rural or highly rural older Veterans with multiple chronic conditions and complex care needs. Clinic staff reported that many of their rural, older Veteran patients were socially and geographically isolated and had no caregiver. Staff also noted that rural, older Veteran patients tended not to be connected digitally via the internet and had difficulty traveling—conditions that compounded these patients’ geographic and social isolation. Table 2 lists the most common social risk factors and gaps in care identified by staff during interviews.

### Rural veterans on the edge of the digital divide

CBOC staff described the circumstances of rural, older Veterans whose access to telemedicine may be negatively impacted by one or more social determinants of health and who, therefore, may be on the edge of the digital divide. These Veterans may lack access to the infrastructure necessary to use telemedicine where they live, such as reliable high-speed internet and adequate cellular network service. For the subset of older, rural Veterans who previously had a degree of technology literacy, they may find their digital knowledge, skills and equipment growing increasingly obsolete, for example, after being out of the workforce due to retirement and as a result of being on a limited income. These challenges may be exacerbated by changes in physical functioning, which can compound the difficulty of using current technologies like tablets and smart phones that require fine motor skills. Table 3 features quotes from CBOC staff interviews highlighting these key barriers. In the last quote in Table 3, a telemedicine technician described the compounding effects of lower levels of digital or computer literacy and declining physical functioning that can occur as we age.

### Convergence of multiple types of isolation characterize the social ecological context of rural, older veterans caught in the digital divide

**Figure 2.**
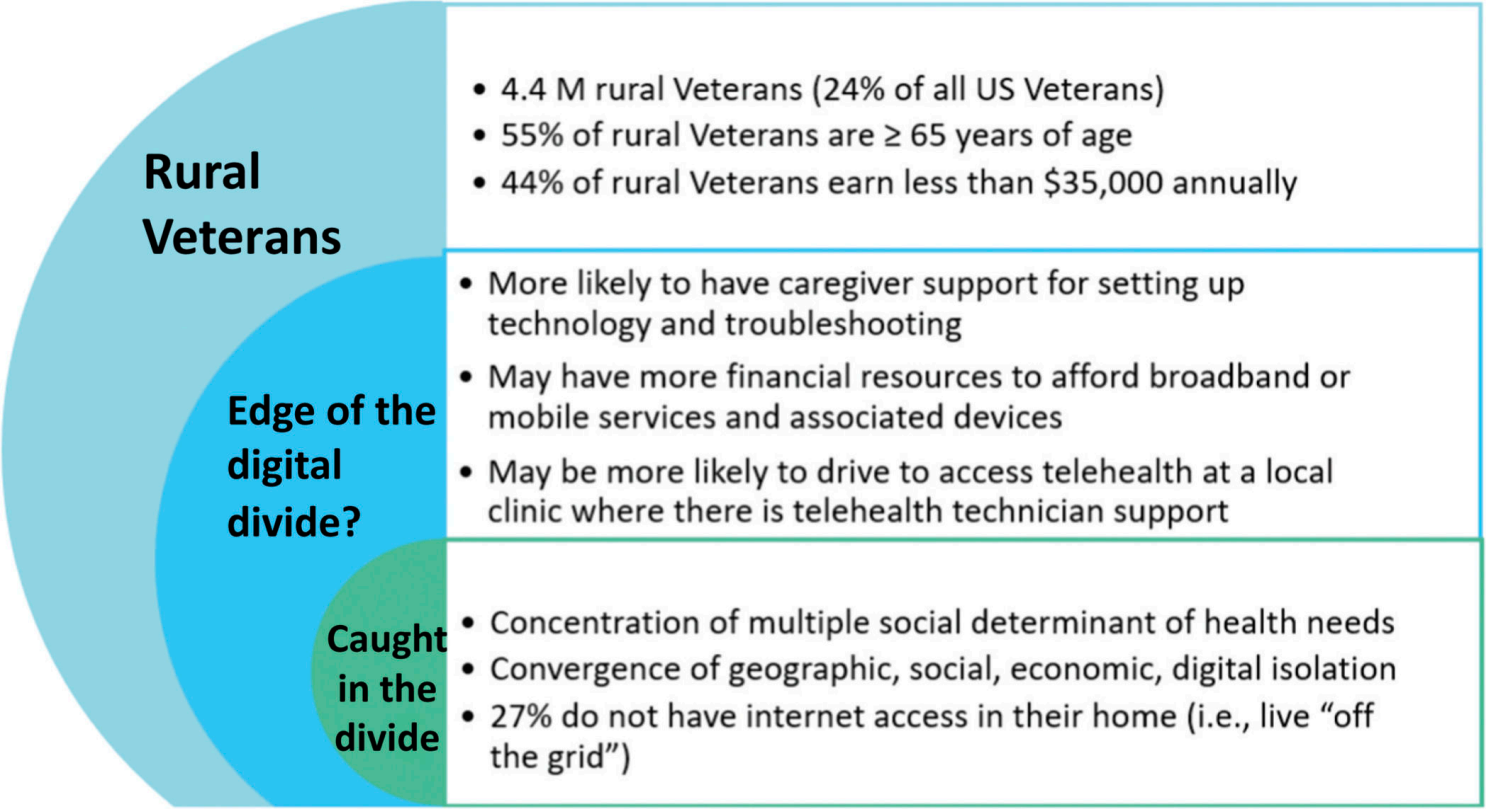
Veterans caught in the digital divide—another social determinant of health of rural, older veterans.

During key informant interviews, CBOC staff described those Veterans whom they could *not* reach effectively, despite multiple attempts and usually by phone. Salient quotes, featured in Table 4, highlight the concentration of different types of isolation—geographic, social, economic and digital isolation—for those rural, older Veterans caught in the digital divide (the population of rural, older Veterans represented in the green segment of the concentric circle diagram shown in Figure 2). During our interviews, rural clinic staff described how very challenging it can be to bridge these divides to reach rural, older Veterans with needed services and support, even by phone. A licensed clinical social worker we interviewed referred to isolation as a ‘meta-influencer’, which constrained choice, perspective and hope and contributed to despondency and self-neglect. The following is an extended excerpt from the interview:

INTERVIEWER: … How does that social and geographic isolation affect their health and healthcare needs?

CBOC Staff: I think it’s a meta influencer … It lays the foundation for everything … I think it’s a major driver of the way they see the world, [such that] they wouldn’t even necessarily, one, recognize that they had a problem or, two, even realize that there was help available to resolve it.

The CBOC staff continued, highlighting that opting for relative geographic and social isolation, for example, to cope with conditions precipitated by military service such as PTSD, can limit awareness of and access to needed healthcare services over time and as one ages.

CBOC Staff: “Lots of people came here to kind of get away from it all when they were younger and healthier. And then they find themselves not doing well with that level of isolation. Not doing well physically, you know, with tangible resource limitations and then also the social isolation … My veterans are very isolated all the time. They’re home-bound pretty much … I think it is sort of one of those things like high blood pressure: its silent and tedious and something operating in the background all the time that has a negative impact.

INTERVIEWER: When you described the level of isolation and that it’s a foundation for their perspective and that they may not be aware that there’s a problem much less a solution, what I imagined is they have no one or nothing to compare their own experience to, so they don’t know what could be better.

CBOC Staff: That [is] exactly what I meant.

Later in the interview, this social worker underscored the importance of partnerships with other community-based organizations as one approach they used to address older, rural Veteran’s unmet needs.

## Discussion

Key informant interviews with CBOC staff highlighted a number of social risk factors that contributed to disparities in digital access among rural, older Veterans. These subjective reports are corroborated by studies conducted by the VA Office of Rural Health, the Federal Communications Commission, and the Purdue University Center for Regional Development, the latter of which applied a novel Digital Divide Index to identify digital disparities. Rural, older Veterans are more likely to have disabilities resulting from their military service and complex care needs related to multiple chronic conditions. Worsening physical, psychological, and cognitive functioning, combined with lack of social support and geographic distance, intensifies both the need for access to geriatric specialty care via telemedicine and the challenges of accessing healthcare through virtual modalities. Though all rural, older Veterans might benefit from accessing GRECC Connect, we learned from CBOC staff that not all segments of the rural, older Veteran population have the ability or desire to access these virtual geriatric specialty care services.

Rural, older Veterans are not a homogeneous population. From our quality improvement work, we began to differentiate those rural, older Veterans who GRECC Connect could potentially serve (i.e. those populations represented in the blue shaded areas of Figure 2) from those the network of GRECC Connect hubs and their CBOC partners were struggling to reach even by phone (i.e. those represented in the green shaded region of Figure 2). A key differentiator was the sheer number of social risk factors(e.g. financial resources, safe shelter, transportation) and the convergence of different types of isolation (e.g. geographic, technological and social). The loss of even one resource might make the difference between a rural, older Veteran having access to telemedicine services versus falling into the digital divide and associated cracks in the healthcare system.

Among those on the edge of the digital divide, we learned that cell phones were the digital device most often used, as opposed to computers or tablets. Yet, interviews with CBOC staff, as well as subsequent interviews with rural, older Veterans and care partners, indicated that cell phones are not optimal for participating in telemedicine appointments due to small screen size (Dryden et al., 2023).

### Efforts to keep those on the edge from falling into the digital divide

One outgrowth of GRECC Connect is Geri-TEC, which was an initiative of GRECC Connect partners to improve access to technology, knowledge and skills among older, rural Veterans and caregivers, with a specific emphasis on reaching isolated communities such as Tribal Nations. To meet these specific aims, Geri-TEC leveraged a VA initiative called the Digital Divide program, which makes internet connectivity and VA-issued, internet-connected iPads available to support Veterans’ access to telemedicine (US Department of Veterans Affairs Office of Connected Care, 2021; US Department of Veterans Affairs VA Telehealth). The VA-issued iPad arrives at the Veteran’s home with a set of instructions that includes a phone number to a help line. Veterans also receive a support call before their first telemedicine appointment to help troubleshoot issues, which provides an opportunity for the Veteran to practice logging into a session as they would the day of their appointment.

**Figure 3.**
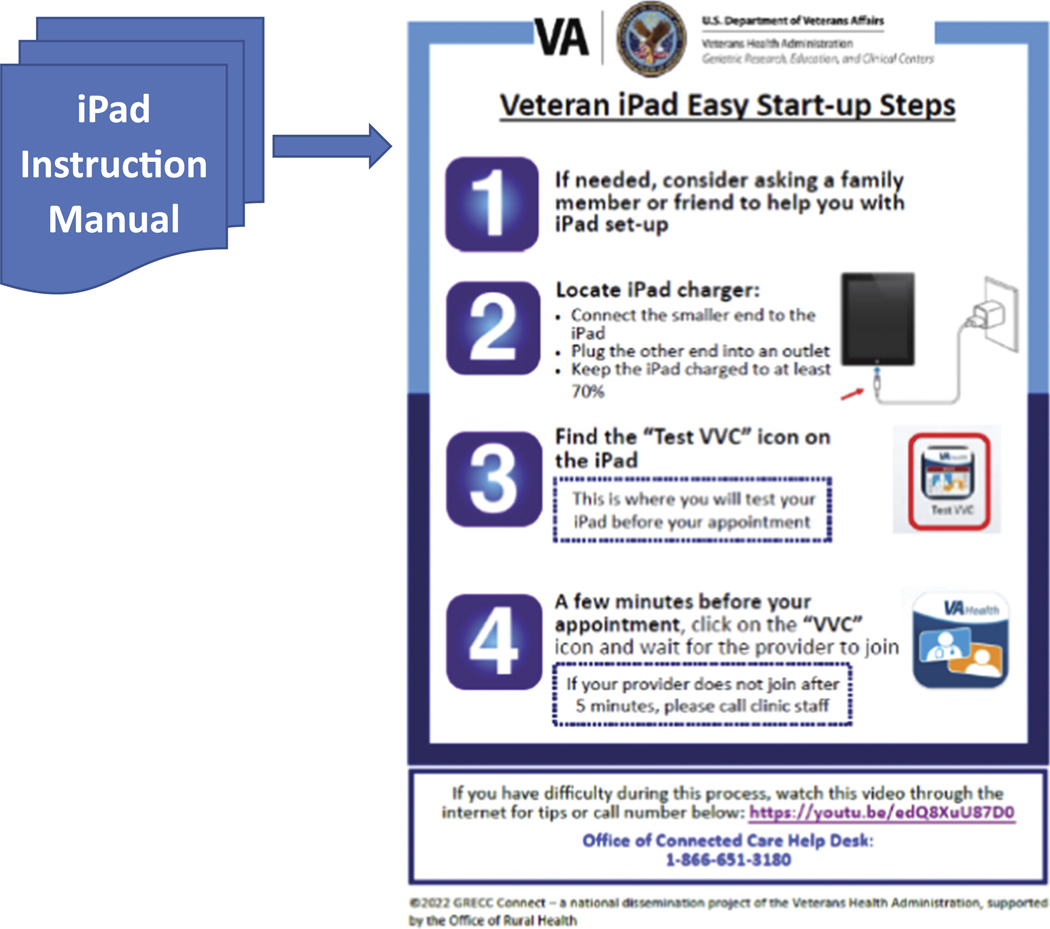
Condensing a 9-page instruction manual into a tip sheet with older veteran input.

**Figure 4.**
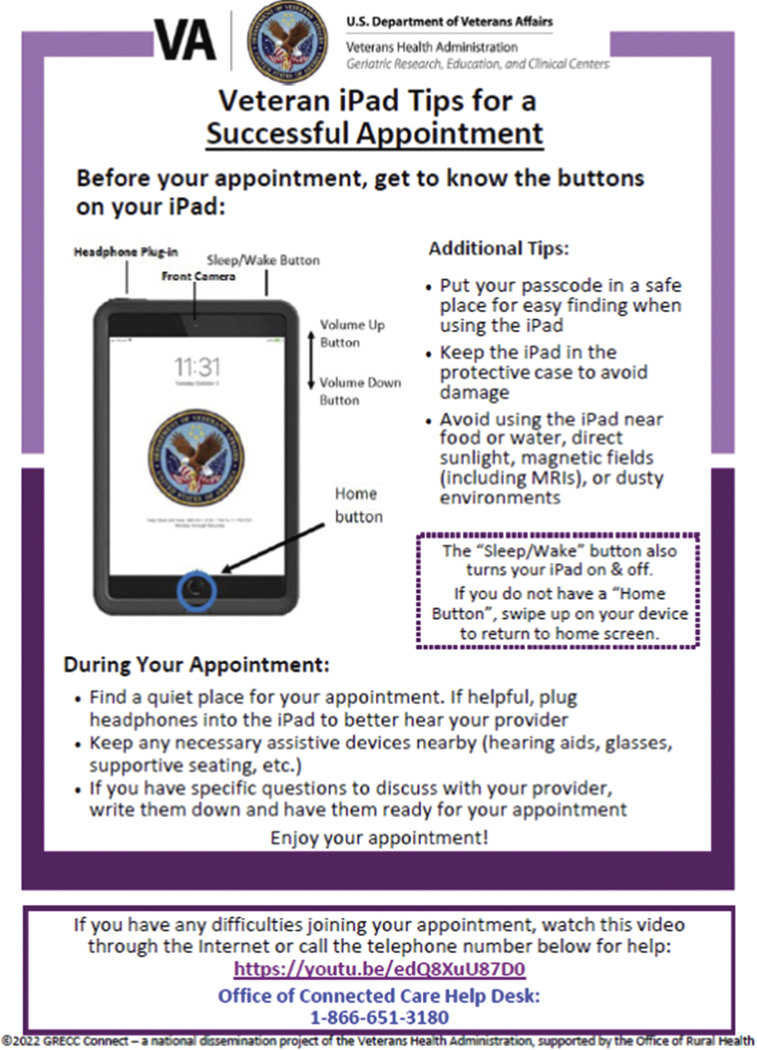
Tip sheet for a successful VA telemedicine appointment.

The VA-issued iPad usually arrives at the Veteran’s home with a 9-page set of instructions in small print that many older users with limited technology experience find overwhelming. With input from the Older Veteran Engagement Team (OVET)—a group of nine older Veterans and one caregiver between the ages of 65–95 who meet monthly to provide feedback on aging-related research and clinical demonstration projects (Nearing et al., 2022), the Geri-TEC team consolidated the information into a one-page Tip Sheet (Figure 3). OVET also suggested that Geri-TEC create a second tip sheet with guidance for older Veterans and caregivers about how to prepare for a telemedicine appointment to optimize the effectiveness of these appointments (Figure 4). OVET members suggested including tips such as logging in several minutes ahead of time to address any technical issues, being in a quiet space free from distractions, writing down questions for the clinician(s) ahead of time, having a notepad available for jotting down notes, and, if possible/appropriate, having a caregiver present who can help listen for and recall important information (H. Lum et al., 2022).

The partnership with other VA programs (in this case the Digital Divide program) and with older Veterans, who have direct lived experience with the challenges and opportunities represented by telemedicine, are key examples of the collaborative approaches needed to support older, rural Veterans on the edge of the digital divide and promote access to geriatric specialty care. While these efforts are critical to supporting digital inclusion, insights from CBOC staff interviews also suggest that such programs will not effectively reach or address the needs of Veterans who are ‘off the grid’ (i.e. without internet connectivity) either by choice or life circumstances. For example, if there is limited or zero 4 G signal where they live, the VA tablet (even with simplified instructions and extra tech support) will not help. Further, research conducted by the Federal Communications Commission suggests that a significant segment of the Veteran population does not see the relevance of or need for digital access—perceptions that affect adoption. The authors reported:

Digital literacy and the perception of relevance of broadband contribute to gaps for those who have not adopted broadband … Many Veteran-led households are more likely to believe they do not need or are not interested in Internet service at home. This pattern can also be observed in older non-Veteran populations, but the tendency of Veterans to be, on average, older than the general population makes age-related adoption barriers more prominent (page 12) (Wireline Competition Bureau Federal Communications Commission USA, 2019).

These data suggest that even Veterans with the financial means may not choose to adopt such technology. Trust, for example, in technology and privacy/security related concerns, may be a salient underlying issue for at least some of these Veterans.

Fulfilling the VA mission of serving all those who served will require diverse approaches that are tailored to specific segments of the older Veteran population. Telemedicine is an important resource supporting older, rural Veterans and caregivers, and GRECC Connect hubs and their CBOC and other programmatic partners are working to prevent rural, older Veterans from falling into the digital divide. However, telemedicine is not a panacea for all rural, older Veteran populations. A nuanced understanding of these populations is necessary to tailor the deployment of a diverse array of VA programs and care delivery modalities, which can include Veteran outreach and engagement by peer specialists.

### Implications for other populations

**Figure 5.**
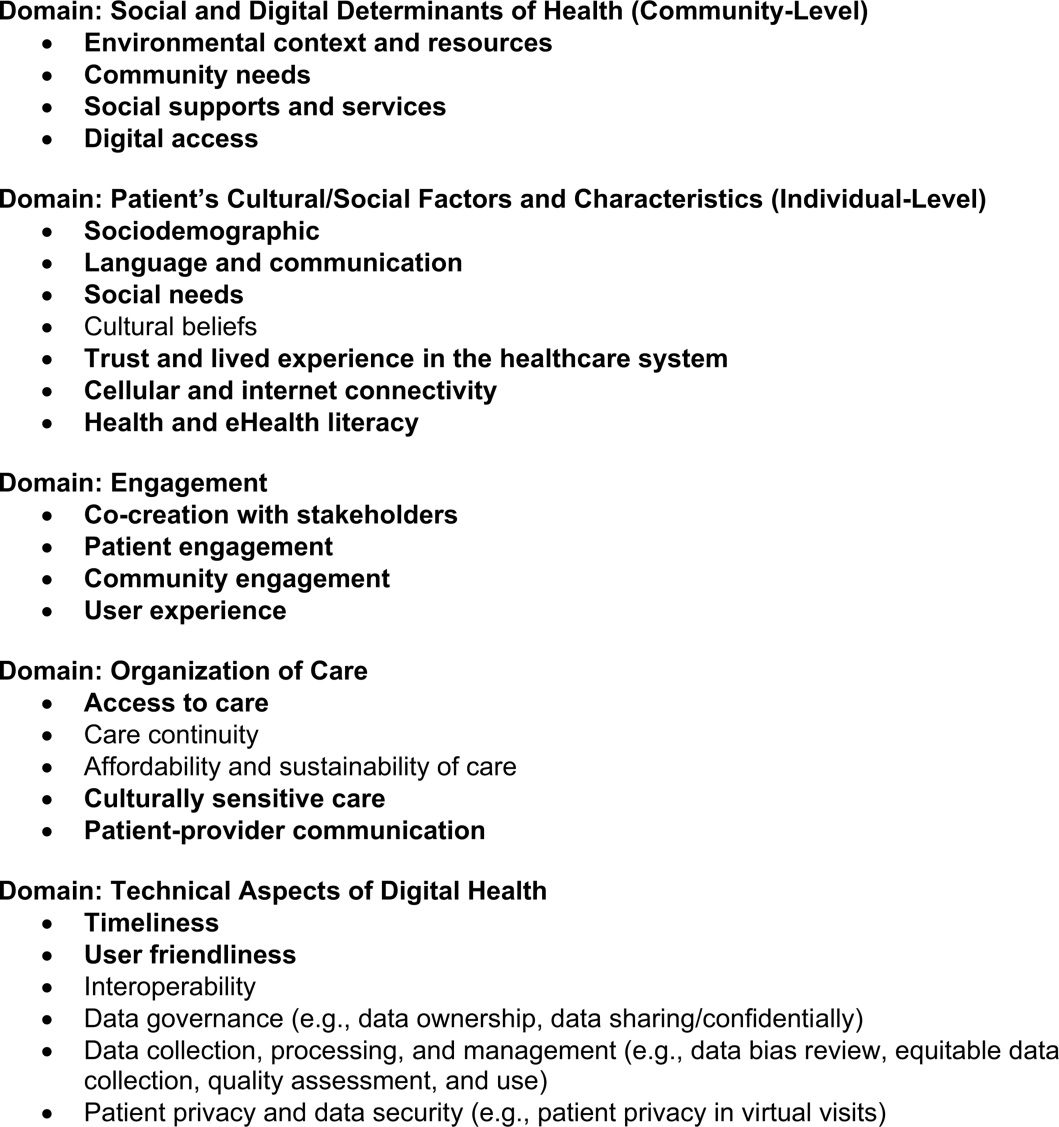
Agency for healthcare research and quality.

Insights emerging from key informant interviews with CBOC staff who serve primarily rural, older Veterans helped us differentiate rural, older Veterans with limited or no access to telemedicine and those who were at risk of having diminished access over time should their circumstances change. Our findings align with the Agency for Healthcare Research and Quality (AHRQ) digital healthcare equity framework (Figure 5) (Hatef et al., 2023). Aspects of the comprehensive framework that are most explicitly congruent with our findings appear in bold-face font in this figure and are explicated here.

Our findings most closely align with the Social and Digital Determinants of Health (Community Level) and Patient’s Cultural/Social Factors and Characteristics (Individual Level) domains. Specifically, observations of rural CBOC staff, triangulated with national data sources, highlight unique characteristics of rural, older Veteran populations that are highly correlated with digital disparities. Particularly salient are the large number of older Veterans who reside in rural or highly rural areas where broadband and cellular access may be limited or unreliable. When compared to the civilian population, rural Veterans are relatively older; have more disabilities; may have had fewer educational opportunities requiring use of digital technologies; and, may have fewer financial resources—all factors that negatively impact digital literacy, access and adoption.

CBOC staff described how lower levels of digital literacy made it difficult to guide new users through the steps of setting up digital devices and logging into a telemedicine appointment (see, for example, the last quote in Table 3 from a telemedicine technician). This technology-related language barrier also came to the fore during the OVET meeting as we workshopped the development of the tip sheets featured in Figures 3 and 4. For example, a caregiver on the team highlighted that many older Veterans may not know what an ‘app’ is.

Our years of working with rural, older Veterans suggest that issues such as PTSD and lack of trust may underlie patterns such as the desire to live in a rural or highly rural area away from densely populated urban centers. This choice to live in more rural areas can constrain digital access. Trust directly influences patterns of adoption and use of the internet and may be a particularly salient issue among those Veterans described as ‘off the grid’. In terms of lived experience with the healthcare system, being required to interface with automated systems (such as the VA’s call center or the VA’s telemedicine platform) may be particularly off-putting for these Veterans. This sentiment was captured by a primary care physician who described Veterans who call into the call center but are intolerant of long wait times (see the last quote in Table 4).

Issues of language (patient-provider communication) and trust negatively impact access to care via telemedicine, which can have downstream consequences affecting timeliness of care and continuity of care, particularly for rural, older Veterans with limited transportation resources and caregiver support. These aspects of rural, older Veteran experiences, as described by CBOC staff, suggest that telemedicine may not be a responsive way to extend access to care for some populations of Veterans. Trust, in particular, and its relationship with digital access and engagement is an important area warranting further investigation in future research.

The Engagement domain of the AHRQ digital healthcare equity framework is salient to our experience of engaging CBOC staff to learn about their experiences working to connect rural, older Veterans to telemedicine for enhanced access to geriatric specialty care. In addition, we engaged older Veterans, who were users of telemedicine or who themselves have had difficulty accessing these services, to inform the development of patient-facing resources for using VA-issued digital devices and having Veteran-centered telemedicine appointments. This is consistent with AHRQ’s conceptualization of the Engagement domain: stakeholder engagement for identifying challenges *and* for co-designing solutions. In summary, the original intent of our study (to identify barriers and facilitators of telemedicine adoption/uptake) and subsequent use of findings (to support ongoing quality and process improvement) is congruent with the AHRQ framework, which encompasses both the root causes of digital disparities, as well as processes that can help change these conditions to achieve digital inclusion.

Above, we suggested further examination of trust as a factor that might influence salient patterns such as the rural-urban geographic distribution of Veteran populations, which determines relative access to high-speed internet and cellular service, as well as patterns in the adoption of digital technologies. Future research may also further develop the concept of digital access as a super determinant of health. Findings in relation to rural, older Veterans on the edge of the digital divide appear consistent with this conceptualization. These Veterans and/or their social networks are actively engaged in trying to access telemedicine to receive needed health information and healthcare services, as well as referrals to community supports that can address other social needs such as transportation, food and housing. A compelling finding from our CBOC staff interviews is that for rural, older Veterans ‘off the grid’, their super determinant of health may, in fact, be isolation—a ‘meta influencer’ with multiple dimensions, including geographic, social, economic, psychological and technological isolation. This new insight underscores the importance of research to explicate the unique conditions underlying telemedicine accessibility, acceptability, and utilization for distinct populations of older, rural Veterans. Investment in such research is essential to guide the development and deployment of digital health solutions, as well as other outreach and engagement approaches to effectively reach and serve all those who served. We very much intend this as a call to action, in keeping with the World Health Organization’s endorsement of digital access as a component of one’s right to health (Grey, 2020; World Health Organization [WHO], 2023).

### Limitations

Interviews with CBOC staff working with Veteran populations in select regions may not reflect all barriers to telemedicine access and utilization given local variations in social determinants of health and social risk factors. Further, for this quality improvement project, we did not collect data directly from rural, older Veterans who are off the grid or otherwise not effectively reached through telemedicine and other healthcare modalities. While the Older Veteran Engagement Team did include one Veteran who does not have access to the internet, their voice/perspectives were sought in designing the tip sheets and did not inform the themes featured in this paper, which emerged solely from CBOC staff interviews.

## Conclusion

The VA has made significant investments to support access to telemedicine for Veteran populations—efforts that preceded and were accelerated by the COVID-19 pandemic. Veteran populations on the edge of the digital divide can be well served by established programs such as GRECC Connect, which leverages interprofessional teams who bring a decade of experience using telemedicine to provide geriatric specialty care to rural, older Veterans. However, there are many other rural, older Veterans who are geographically, socially, economically and digitally isolated and are not effectively reached by this and other programs the VA has established to bridge the digital divide. Attempts to bridge the digital divide for this population are limited by infrastructure/broadband availability even with VA-provided tablets; those with low technology literacy and without family or community supports may not benefit from VA programming targeting the digital divide. If this is true within the VA, given the investment of funding, programming, equipment, staff training and technical support, there must be many more civilian populations who do not qualify for these supports but need them. The implication is that the digital divide may only grow, particularly given the growing aging population nationally and globally. Key insights from this work underscore the importance of studies to explore the unique contextual factors and environmental conditions that shape accessibility, acceptability and utilization of telemedicine among different populations of older adults. This understanding is essential to support access to healthcare as a human right in the digital era.

## Figures and Tables

**Table 1.** Demographic characteristics of rural, older veterans highly correlated with disparities in digital access.

Characteristics correlated with disparities in digital access (DDI)	Demographics of Rural Veterans
Age	● Rural Veterans enrolled in VA’s health care system are significantly older [than urban Veterans]: 55 percent are over the age of 65^a^
Disabilities	● 58 percent of rural enrolled Veterans have at least one service connected condition^a^ ● [The older, rural] Veteran population is medically complex and more likely to be diagnosed with diabetes, obesity, high blood pressure and heart conditions that require more frequent, ongoing care^a^
Educational Attainment	● A higher percentage of rural Veterans (44%) compared to urban Veterans (33%) have completed less than a high school degree^c^
Infrastructure (Built Environment)	● 27 percent do not access the internet at home^a^
Socio-economic status	● 44 percent earn less than $35,000 annually^a^ ● 7% live in poverty^b^
**Other Variables Identified by** Darrat et al. (2021)	**Demographics of Rural Veterans**
Gender	● 92 percent of enrolled rural Veterans are men^a^
Marital/Partnered status	Information pertaining to the general Veteran population (not specific to rural Veterans): ●‘Veterans are more likely to be men and live alone than non-Veterans … Household demographics have an effect on fixed and mobile subscriptions among Veterans. As Veterans are more likely to be men living alone, they also tend to have the lowest subscription rates to fixed and mobile broadband’^d^ ● Studies consistently highlight the importance of caregivers^a^ to older Veterans’ ability to access telemedicine^e,fb^

a https://www.ruralhealth.va.gov/aboutus/ruralvets.asp#:~:text=VA%20recognizes%20the%20need%20to,budget%20to%20rural%20Veteran%20care. Accessed December 26, 2023.

b https://www.census.gov/library/publications/2017/acs/acs-36.html. Accessed December 26, 2023.

c U.S. Census Bureau, American Community Survey, 2014 Prepared by the National Center for Veterans Analysis and Statistics. Accessed December 26, 2023.

d Federal Communications Commission USA. Report on Promoting Broadband Internet Access Service for Veterans, Pursuant to the Repack Airwaves Yielding Better Access for Users of Modern Services Act of 2018. Prepared by the Wireline Competition Bureau. Submitted to the Senate Committee on Commerce, Science, and Transportation House of Representatives Committee on Energy and Commerce. May 2019.

e Gately ME, Waller D, Metcalf EE, Moo LR. Occupational Therapy Practitioner Perspectives of the Role of Caregivers in Video Telehealth. J Gerontol Nurs. 2022 Oct;48(10):15–20. doi: 10.3928/00989134-20220908-02. Epub 2022 Oct 1. PMID: 36169296; PMCID: PMC9577539.

f Dryden EM, Kennedy MA, Conti J, et al. Perceived benefits of geriatric specialty telemedicine among rural patients and caregivers. Health Serv Res. 2023; 58(Suppl. 1): 26–35. doi:10.1111/1475-6773.14055.

**Table 2.** Social risk factors and gaps in care of rural, older veterans identified by CBOC staff (listed alphabetically in each column).


● Cardiology	● Home-based care	● Pulmonology
● Caregiver respite, resources	● Mental health care	● **Technology/connectivity**
● Dementia diagnosis, care	● Outreach/education	● Transition to long term care
● Food insecurity	● Palliative Care	● Transportation services


**Table 3.** Quotes from CBOC staff highlighting contextual factors that leave rural, older veterans on the edge of the digital divide.


Infrastructure	‘I have **Veterans** that in theory **have the technology** and they **know how to use it**, but their internet is not strong enough … So, I have not been able to do any video visits … The **lack of high-speed internet** in our area **is huge**’. (Psychologist)
Access to technology	‘It seems that it’s really hard for a lot of them to access virtual care other than just the phone. I don’t know if that’s … because **they don’t have the technology or the familiarity with it**’. (Psychologist)
Digital literacy combined with physical limitations	‘You’re asking an 80-year-old man to not only **navigate** an **email** but **navigate an iPad**. “**Click here, click there**”. Some might have **Parkinson’s**. Some might have **hearing loss**. Some have **sight issues**. We’re trying to make everything more acceptable to them, but we’re also [asking] them to do something that is **hard for them** and **not familiar** and that they’re **not comfortable** with’. (Telemedicine Technician)


**Table 4.** Convergence of multiple types of isolation and other social risk factors as the social ecological context of rural, older veterans caught in the digital divide.


Off the Grid	● ‘They live in tiny communities that branch out on the peninsula with 60 to 70 miles [in] between … Many of these patients live up in the mountains, or they live in areas that are **very off the grid**. **They don’t have computer access or cellphone access**’. (Clinic Manager) ● **‘Older Veterans—there are a number of them, especially again with PTSD, … they’re living off the grid**. If they had local places where they could just drop in and connect for basic needs, medical needs and mental health needs, that would be amazing’. (Social Worker)
Lack of safe shelter/ housing	● ‘We also have a very large **homeless** population, unfortunately, living in the area that **live** … **in the trees** and, … **in little caves**, trying to survive out here’. (Clinic Manager)
Intersection-ality (Geography, Age, SES)	● ‘A lot of our **more rural patients** … **don’t tend to have on-line access** … **Many are very poor** and almost homeless or **on the edge of homeless**, **living in trailers out on little pieces of land …** By the time they call into the Call Center and sit on hold for five or ten or 20 minutes … [or we] try to call them back and **get through to them**, it’s **not easy even when we do finally make contact**. **That system isn’t working well for that population**. The younger patients are more savvy on computers and cellphones, and they seem to be much more reachable, and they may have more financial means, too; that may be part of it’. (Primary Care Physician)


## Data Availability

The data that support the findings of this study are available from the corresponding author (KAN) upon reasonable request.

## Appendix A: Glossary of Terms

### Community-based Outpatient Clinic (CBOC):

A VA clinic, separate from an urban-based VA medical center (hospital), that provides the most common outpatient healthcare services closer to where rural Veterans live. Services vary by CBOC but typically include primary care, mental health care, treatment for acute conditions and management of chronic healthcare conditions. CBOCs are typically staffed with a primary care clinician, pharmacist, psychologist, social worker, nurse professional, telehealth technician and clinic manager. Telemedicine is used to extend access to specialty care not available onsite (source: https://www.va.gov/health/aboutvha.asp#:~:text=Community%2DBased%20Outpatient%20Clinic%3A%20To,visiting%20a%20larger%20medical%20center).

### Digital divide:

disparities in access to adequate broadband infrastructure, reliable internet connectivity, up-to-date technology equipment, and digital or computer literacy, with disparities impacted by health conditions (mental health, physical and cognitive functioning), age, geography, and education and job opportunities. (resource: Bridging the Digital Divide | Telehealth VA)

### “Off the Grid”:

While historically used to refer to those who were not connected to public utilities, Community-based Outpatient Clinic staff used this term during key informant interviews to refer to patients who did not have access to, or wish to use, an internet or email service provider and associated equipment, because they did not trust it, see the need for it, could not afford it and/or did not have access to the infrastructure to support it. CBOC staff noted that these patients often lived in remote areas, experienced housing instability and were challenging to reach even by phone. Further elaborating on the distaste for technology in general, CBOC staff noted that these Veterans were not tolerant of automated systems, such as the VA call center used to manage call volume.

### Service-Connected Disability:

A health condition resulting from military service. Veterans with verified service-connected disabilities receive a service-connected disability rating based on the type(s) and severity of the disability(s). The service-connected disability rating determines the corresponding monthly compensation and eligibility for other benefits. (source: https://www.va.gov/disability/about-disability-ratings/)

### Social Determinants of Health:

The World Health Organization defines social determinants of health as the non-medical factors that influence health. They are the “conditions in which people are born, grow, work, live, and age, and the wider set of forces and systems shaping the conditions of daily life [including distribution of resources and opportunities] … Research shows that the social determinants can be more important than health care or lifestyle choices in influencing health.” (source: Social determinants of health (who.int))

### Social Needs:

Social risk factors that patients have prioritized to address, for example, through the engagement of community resources and supports. Social needs is a term that reflects patient priorities, preferences and choice to optimize the effectiveness of social interventions. (source: Meanings and Misunderstandings: A Social Determinants of Health Lexicon for Health Care Systems | Milbank Quarterly | Milbank Memorial Fund)

### Social Risk Factors:

Individual-level adverse social determinants of health or specific adverse social conditions associated with poor health, like social isolation or housing instability. (source: Meanings and Misunderstandings: A Social Determinants of Health Lexicon for Health Care Systems | Milbank Quarterly | Milbank Memorial Fund)

## Appendix B: CBOC Key Informant Interview Guide for GRECC Connect Quality

**Improvement Project** (originally published in Pimentel CB, Dryden EM, Nearing KA, et al. The role of Department of Veterans Affairs community-based outpatient clinics in enhancing rural access to geriatrics telemedicine specialty care. *J Am Geriatr Soc*. 2023; 1–9. doi:10.1111/jgs.18703)

Script: *Thank you for talking with us today. We are working with the VA Office of Rural Health to gather information from CBOCs on their experience supporting older Veterans and caregivers in rural communities with multiple chronic conditions and complex care needs.*

The interview will explore geriatric specialty needs and current practices, ways your CBOC may be using geriatric specialty consultation telehealth services, facilitators, and barriers to using these services, and any perceived benefits. Do you have any questions about our purpose?

Your participation is entirely voluntary. Each interview will take approximately 30–60 minutes. We will be conducting multiple interviews with staff at this CBOC. We are planning to talk with more than 60 people across the VA and will maintain the confidentiality of individuals. We will provide a formal report summarizing key themes that emerge across all interviews for the Office of Rural Health.

To accurately record the information you share with us, we would like permission to audio-record our discussion. If you do not want us to audio-record our conversation, you can still take part in the conversation. I will just need some time to take notes while we talk.

Are there any questions before we begin?

[Turn on the recorder]

“This is [your name] and today’s date is [__] and I’m conducting an interview with the (title/role). Do I have your permission to record our interview?” ( ) Yes ( ) No 1

### Introduction

1. I would like to start by learning a little more about you.
  - How long have you worked at this CBOC and been in your current role as (title/role)?
  - What are your main responsibilities?
  - Please tell me about your experience at the VA or elsewhere that involved working with rural and/or geriatric patients?

#### Geriatric Population and Needs

With the following questions, I’d like us to focus on older, rural Veterans with multiple chronic conditions and complex care needs.

1. What proportion of the patient population served by this CBOC would you say match this description?
  - What are their most pressing needs?
  - What resources/services do you currently provide to address the needs of this geriatric population, including caregivers? (Probe: What do you feel comfortable/have the capacity to manage or coordinate?)
  - What resources/services do you have available in the community (outside the VA) that you typically connect older Veterans and caregivers to?
  - What are the current gaps in available resources/services to meet the needs of your older, complex rural Veterans and their caregivers? (Probe: What are your biggest challenges in caring for older rural Veterans with multiple chronic conditions?)
  - What would be helpful to have from another team, VA or otherwise?
2. If you think about rural, older Veterans, what segments of this patient population do you feel you are serving very well?
3. Are there any specific rural geriatric populations you have trouble serving/reaching as well as you would like to? (Probe: Those with dementia? Veterans with palliative care needs? Homeless older Veterans? Older women veterans? Older Native American veterans?)

#### Geriatric Specialty Consultations: General

Now we are going to talk specifically about geriatric specialty consultations:

1. How often do you/staff at this CBOC seek geriatric specialty consultations for your rural patients?
  - *If they say never or not very often*: Can you tell me more about that? (Probe, if necessary: Is that because of the population you serve or because of the expertise you have in-house?)
  - *If they say they do access them and have not already answered this above*: How do you currently access a geriatric specialty consultation?
  - What might prompt a geriatric specialty consultation for a specific patient/case?
  - Do you feel you/the staff here has sufficient access to geriatric specialty consultations when needed to care for patients? Please explain.

#### Familiarity with GRECC-Connect/General Use

Next I would like to move on to talk about the GRECC Connect program services.

One of the programs that the Office of Rural Health supports is called GRECC Connect. Is that name familiar to you? *[Normalize that it is ok not to be familiar as the VA has so many programs, etc.]* Among other things, this program supports access to general and specialty geriatric care through telehealth clinics and e-consultations. Do the services sound familiar even if the name does not?

(*Here is where the interviews would diverge if they are still not familiar at all with the program.* ***If not familiar, skip to question 17***)

1. For those that ARE familiar with these services:
  - What is your experience with [these telehealth clinics and related e-consultations] here at this CBOC?
  - How did you first learn about GRECC Connect [these services]? (Probe, if necessary: How was it introduced to your site e.g. encouraged by leadership/requested by staff? What types of training were provided?)
  - Which services are used, if any, at this CBOC? (Probe, if necessary: To what extent are they used? Episodic or routine engagement? Widespread use? Anyone who uses these services regularly, i.e. champions?)

#### Ease of Use

1. Thinking back to a time when you tried to use or used the GRECC Connect services, can you describe what that experience was like? Does this reflect your experience with these services, in general?
2. In general,
  - What factors have helped to make the use of GRECC Connect successful here? (Probe: relationship with GRECC Connect clinicians e.g. comfort level working with them, mutually respectful collaboration?)
  - What factors have made the use of GRECC Connect challenging here? i.e. what are the barriers preventing you from using these services? (Probe: relationship with GRECC Connect clinicians e.g. comfort level working with them, mutually respectful collaboration?)
  - In what ways have you adapted the processes to make it work better for your site?

#### Perception of Services

1. To what extent are GRECC Connect services aligned with what you feel this CBOC’s patients and staff need? (Probe about their perceptions of GRECC Connect’s value and usefulness in helping to address the challenges of caring for older rural Veterans with multiple chronic conditions and complex care needs?)
2. What are the advantages of using GRECC Connect services? a. In the absence of these services, what would you do? What resources might you tap into?

#### Outcomes

Now I would like to shift focus a bit to try to understand more about potential impacts this program may have.

1. In terms of how this program may have impacted you as an individual, I want to start by asking: What are your biggest sources of stress related to caring for older, rural Veterans with complex care needs?
  - In that context, I would like to ask you to reflect on how this program (GRECC Connect) has affected how you feel about your work and your ability to care for older Veterans? (Probe, if necessary: your feelings of being supported, your workload, job satisfaction, or feelings of burnout?)
2. In what ways has this program impacted your CBOC’s rural geriatric patients? (Probe, if necessary: quality of life, reduction in quantity or severity of symptoms, reduced need for community/non-VA care, fewer trips to main VAMC)
3. In what ways has this program impacted your CBOC’s rural geriatric patients’ caregivers? (Probe, if necessary: time savings, cost reductions, reduced fatigue)

#### Recommendations/Final Thoughts

1. What more could GRECC Connect do to help you serve your rural geriatric patients? (Probe: What are the gaps/most important unmet needs for these patients? Caregivers? Clinicians?)
2. What recommendations would you make to those trying to provide these services and coordinate with CBOCs? What would make it easier?
3. Anything that we haven’t discussed today about the needs of your rural geriatric patients/caregivers/clinicians and GRECC Connect that you feel we should know?

Thank you!

For those that are **NOT** familiar with GRECC Connect:

1. Now that you know about GRECC Connect services, in what ways, if any, do you think these services could help address the needs of rural geriatric patients?
2. How do you think GRECC Connect services would need to be introduced and implemented in order to support this CBOC’s clinicians?
3. Do you foresee any barriers or difficulties with implementing GRECC Connect services at your CBOC?
4. If you could use these services, what impacts do you think it could have on:
  - Your rural geriatric patients and their caregivers?
  - On you? (Probe, if necessary: on their feelings about their work, on their ability to care for older Veterans with complex conditions)
5. What recommendations would you make to those providing GRECC Connect services and coordinating with CBOCs?
6. What other types of resources/services would help you meet the needs of your rural geriatric patients, their caregivers, and clinicians?
7. Anything that we haven’t discussed today about the needs of your rural geriatric patients/caregivers/clinicians and the GRECC Connect program that you feel we should know?

Thank you!

## References

[R1] Clare CA (2021). Telehealth and the digital divide as a social determinant of health during the COVID-19 pandemic. Network Modeling Analysis in Health Informatics and Bioinformatics, 10(1), 26. 10.1007/s13721-021-00300-y 33842187 PMC8019343

[R2] County Health Rankings and Roadmaps. (2021, December 14). Webinar: Broadband: A Super Determinant of Health. https://www.countyhealthrankings.org/online-and-on-air/webinars/broadband-a-super-determinant-of-health

[R3] Darrat I, Tam S, Boulis M, & Williams AM (2021). Socioeconomic disparities in patient use of telehealth during the coronavirus disease 2019 surge. JAMA Otolaryngology Head & Neck Surgery, 147(3), 287–295. 10.1001/jamaoto.2020.5161 33443539 PMC7809608

[R4] Dhanani Z, Ferguson JM, van Campen J, Slightam C, Jacobs JC, Heyworth L, & Zulman D (2023). Overcoming access barriers for veterans: Cohort study of the distribution and use of veterans affairs’ video-enabled tablets before and during the COVID-19 pandemic. Journal of Medical Internet Research, 25, e42563. Retrieved January 26, 2023. from, 10.2196/42563. PMC9912147 36630650

[R5] Dryden EM, Kennedy MA, Conti J, Boudreau JH, Anwar CP, Nearing K, Pimentel CB, Hung WW, & Moo LR (2023). Perceived benefits of geriatric specialty telemedicine among rural patients and caregivers. Health Services Research, 58(Suppl. 1), 26–35. 10.1111/1475-6773.14055 36054487 PMC9843069

[R6] Federal Communications Commission. (2022, February 7). Advancing Broadband Connectivity as a Social Determinant of Health. https://www.fcc.gov/health/SDOH

[R7] Gale NK, Heath G, Cameron E, Rashid S, & Redwood S (2013). Using the framework method for the analysis of qualitative data in multi-disciplinary health research. BMC Medical Research Methodology, 13(1), 117. 10.1186/1471-2288-13-117 24047204 PMC3848812

[R8] Gallardo R (2022, August 17). The State of the Digital Divide in the United States. Purdue University Center for Regional Development. https://pcrd.purdue.edu/the-state-of-the-digital-divide-in-the-united-states/

[R9] Gallardo R (2023, February 1). 2021 Digital Divide Index (DDI). ArcGIS StoryMaps. https://storymaps.arcgis.com/stories/8ad45c48ba5c43d8ad36240ff0ea0dc7

[R10] Geriatric Scholars Virtual Learning Community. (2023, December 29). GRECC Connect. https://www.gerischolars.org/mod/page/view.php?id=1066

[R11] Grey A (2020, December 10). The case for connectivity, the new human right. United Nations UN Chronicle. https://www.un.org/en/un-chronicle/case-connectivity-new-human-right

[R12] Hatef E, Austin JM, Mills C, Wiley K, Hwee T, Scholle SH (2023, June). Environmental scan to determine the Current state of existing, related frameworks and best practices for creating equitable healthcare solutions involving digital technologies. (prepared by Johns Hopkins University under contract No. 75Q80120D00015). AHRQ publication No. 23–0028. Agency for Healthcare Research and Quality.

[R13] Hung WW, Rossi M, Thielke S, Caprio T, Barczi S, Kramer BJ, Kochersberger G, Boockvar KS, Brody A, & Howe JL (2014). A multisite geriatric education program for rural providers in the veteran health care system (GRECC-Connect). Gerontology & Geriatrics Education, 35(1), 23–40. 10.1080/02701960.2013.870902 24397348

[R14] Landi H (2020, June 25). VA Has Spent $39M to Support Telehealth Amid COVID-19. Veterans Say there’s Still a Digital Divide. Fierce Healthcare. https://www.fiercehealthcare.com/tech/va-has-spent-39m-to-support-telehealth-services-amid-covid-19-pandemic-but-digital-divide#:~:text=Veterans%20say%20there’s%20still%20a%20digital%20divide,-By%20Heather%20Landi&text=Like%20other%20health%20providers%2C%20the,during%20the%20COVID%2D19%20pandemic

[R15] Leung LB, Yoo C, Chu K, O’Shea A, Jackson NJ, Heyworth L, & Der-Martirosian C (2023). Rates of primary care and integrated mental health telemedicine visits between rural and urban veterans affairs beneficiaries before and after the onset of the COVID-19 pandemic. JAMA Network Open, 6(3), e231864. Retrieved March 1, 2023, from 10.1001/jamanetworkopen.2023.1864 PMC9993180 36881410

[R16] Lum H, Kelley L, Nearing K, Walker L, Johnson G, Barczi S (2022). Improving video device usage among older rural veterans with user-centered design. Innovation in Aging, 6(Suppl 1), 598. Retrieved December 20, 2022, from 10.1093/geroni/igac059.2235

[R17] Lum HD, Nearing K, Pimentel CB, Levy CR, & Hung WW (2020). Anywhere to anywhere: Use of telehealth to increase health care access for older, rural veterans. Public Policy & Aging Report, 30(1), 12–18. 10.1093/ppar/prz030 40979974 PMC12447726

[R18] Nearing KA, Adams HM, Alsphaugh J, Douglas SE, Feller TR, Fleak R, Moore V, Martin-Sanders S, Schultz TM, Stratton K, Sullivan JP, van Sickle L, Yates JD, Yates TA, & Matlock DD (2022). Engaging the wisdom of older veterans to enhance VA healthcare, research, and services. Journal of General Internal Medicine, 37(Suppl 1), 22–32. 10.1007/s11606-021-07076-x 35349016 PMC8960672

[R19] Pimentel CB, Dryden EM, Nearing KA, Kernan LM, Kennedy MA, Hung WW, Riley J, & Moo LR (2023). The role of department of veterans affairs community-based outpatient clinics in enhancing rural access to geriatrics telemedicine specialty care. Journal of the American Geriatrics Society, 72(2), 1–9. 10.1111/jgs.18703 38032320

[R20] Pimentel CB, Gately M, Barczi SR, Boockvar KS, Bowman EH, Caprio TV, Colón-Emeric CS, Dang S, Espinoza SE, Garner KK, Griffiths PC, Howe JL, Lum HD, Markland AD, Rossi MI, Thielke SM, Valencia-Rodrigo WM, Moo LR, & Hung WW (2019). GRECC connect: Geriatrics telehealth to empower health care providers and improve management of older veterans in rural communities. Federal Practitioner: For the Health Care Professionals of the VA, DoD, and PHS, 36(10), 464–470. PMID: 31768097; PMCID: PMC6837335. 31768097 PMC6837335

[R21] Ritchie J, & Spencer L (2022). Qualitative data analysis for applied policy research. In Huberman A & Miles B (Eds.), The qualitative researcher’s companion (pp. 305–329). SAGE Publications. 10.4135/9781412986274

[R22] Sieck CJ, Sheon A, Ancker JS, Castek J, Callahan B, & Siefer A (2021). Digital inclusion as a social determinant of health. NPJ Digital Medicine, 4(1). 10.1038/s41746-021-00413-8 PMC7969595 33731887

[R23] Tisdale R, Der-Martirosian C, Yoo C, Chu K, Zulman D, & Leung L (Retrieved online January 22, 2024). Disparities in video-based primary care use among veterans with cardiovascular disease. Journal of General Internal Medicine, 39(S1), 60–67. 10.1007/s11606-023-08475-y 38252244 PMC10937859

[R24] Turcios Y (2023, March 22). Digital Access: A Super Determinant of Health. SAMHSA Blog. https://www.samhsa.gov/blog/digital-access-super-determinant-health

[R25] U.S. Department of Veterans Affairs Veterans Health Administration Office of Rural Health. Office of Rural Health Thrive 2020 Annual Report. https://www.ruralhealth.va.gov/docs/ORH0285_2020_ORH_Annual_Report-FINAL_508c.pdf

[R26] US Department of Agriculture Economic Research Service. (2023, September 25). Rural-urban commuting area codes. https://www.ers.usda.gov/data-products/rural-urban-commuting-area-codes/

[R27] US Department of Veterans Affairs. Department of Veterans Affairs FY 2018 – 2024 Strategic Plan . https://www.jcs.mil/Portals/36/Documents/Doctrine/Interorganizational_Documents/dva_strategicplan2018_2024.pdf

[R28] US Department of Veterans Affairs Office of Connected Care. (2021 September). Connecting Veterans to Telehealth Care. https://connectedcare.va.gov/sites/default/files/telehealth-digital-divide-fact-sheet.pdf

[R29] US Department of Veterans Affairs Office of Rural Health. (2023, July 11). Rural Veterans. https://www.ruralhealth.va.gov/aboutus/ruralvets.asp#:~:text=Almost%20a%20quarter%20of%20all,to%20reside%20in%20rural%20communities

[R30] US Department of Veterans Affairs VA Telehealth. Bridging the Digital Divide. https://telehealth.va.gov/digital-divide

[R31] Washington State Department of Health. (2016, October 27). Guidelines for Using Rural Urban Classification Systems for Community Health Assessment. https://doh.wa.gov/sites/default/files/legacy/Documents/1500//RUCAGuide.pdf

[R32] Wireline Competition Bureau. Federal Communications Commission USA. (2019, May). Report on promoting broadband internet access service for veterans, pursuant to the repack airwaves yielding better access for users of modern services act of 2018. Submitted to the Senate Committee on Commerce, Science, and Transportation and House of Representatives Committee on Energy and Commerce.

[R33] World Health Organization. (2023, December 1). Human Rights https://www.who.int/news-room/fact-sheets/detail/human-rights-and-health

